# Glycan and Protein Analysis of Glycoengineered Bacterial *E. coli* Vaccines by MALDI-in-Source Decay FT-ICR Mass Spectrometry

**DOI:** 10.1021/acs.analchem.1c04690

**Published:** 2022-03-16

**Authors:** Simone Nicolardi, Renzo Danuser, Viktoria Dotz, Elena Domínguez-Vega, Ali Al Kaabi, Michel Beurret, Chakkumkal Anish, Manfred Wuhrer

**Affiliations:** † Center for Proteomics and Metabolomics, Leiden University Medical Center, Albinusdreef 2, 2333 ZA Leiden, The Netherlands; ‡ Janssen Vaccines AG (Branch of Cilag GmbH International), Rehhagstrasse 79, CH-3018 Bern, Switzerland; § Bacterial Vaccine Discovery & Early Development, Janssen Vaccines and Prevention B.V., Archimedesweg 4-6, 2333 CN Leiden, The Netherlands

## Abstract

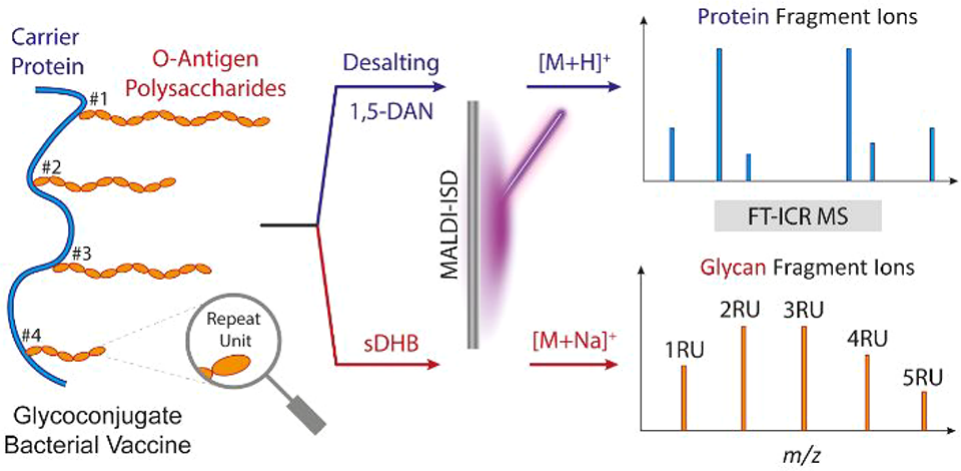

Bacterial glycoconjugate vaccines have a major role in preventing microbial infections. Immunogenic bacterial glycans, such as O-antigen polysaccharides, can be recombinantly expressed and combined with specific carrier proteins to produce effective vaccines. O-Antigen polysaccharides are typically polydisperse, and carrier proteins can have multiple glycosylation sites. Consequently, recombinant glycoconjugate vaccines have a high structural heterogeneity, making their characterization challenging. Since development and quality control processes rely on such characterization, novel strategies are needed for faster and informative analysis. Here, we present a novel approach employing minimal sample preparation and ultrahigh-resolution mass spectrometry analysis for protein terminal sequencing and characterization of the oligosaccharide repeat units of bacterial glycoconjugate vaccines. Three glycoconjugate vaccine candidates, obtained from the bioconjugation of the O-antigen polysaccharides from *E. coli* serotypes O2, O6A, and O25B with the genetically detoxified exotoxin A from *Pseudomonas aeruginosa*, were analyzed by MALDI-in-source decay (ISD) FT-ICR MS. Protein and glycan ISD fragment ions were selectively detected using 1,5-diaminonaphtalene and a 2,5-dihydroxybenzoic acid/2-hydroxy-5-methoxybenzoic acid mixture (super-DHB) as a MALDI matrix, respectively. The analysis of protein fragments required the absence of salts in the samples, while the presence of salt was key for the detection of sodiated glycan fragments. MS/MS analysis of O-antigen ISD fragments allowed for the detection of specific repeat unit signatures. The developed strategy requires minute sample amounts, avoids the use of chemical derivatizations, and comes with minimal hands-on time allowing for fast corroboration of key structural features of bacterial glycoconjugate vaccines during early- and late-stage development.

## Introduction

The emergence and spreading of antimicrobial-resistant (AMR) pathogenic bacteria pose a threat to healthcare systems and economies worldwide.^1^ In the United States (US) alone, more than 23,000 deaths a year are associated with AMR pathogens with an annual healthcare cost of more than $4.6 billion.^2,3^ Globally, AMR infections could lead to 10 million deaths a year by 2050. Therefore, novel therapeutic and prevention strategies are urgently needed to effectively manage AMR infections.^4,5^

Vaccination against pathogenic bacteria prevents bacterial infections and limits the use of antibiotic treatments thus reducing the occurrence of antimicrobial resistance.^6^ Several bacterial vaccines are currently available to tackle bacterial infections, such as meningococcal and pneumococcal diseases, diphtheria, tetanus, and pertussis, and more are under development.^6−8^ Bacterial vaccines rely on a range of different technologies and designs.^6,9−11^ Glycoconjugate vaccines are made of a carbohydrate moiety, derived from either a bacterial capsule or an O-antigen polysaccharide (O-PS), and a carrier protein.^12^ The chemical conjugation of these components requires multiple steps of purification, detoxification, and chemical activation. In contrast, bioconjugation—the recombinant, enzymatic production of a glycoconjugate directly in bacterial cells—is a more straightforward and low-cost method for the production of relatively pure glycoconjugate vaccines.^13^ The coupling of the biosynthesized bacterial polysaccharides and the carrier protein is carried out in bacterial host cells, usually, *Escherichia coli*, using specific enzymes such as the oligosaccharyltransferase PglB.^14−17^ This technology has been recently used to develop glycoconjugate vaccine candidates against *E. coli* serotypes O1, O2, O6A, and O25B by coupling the serotype-specific O-antigens to exotoxin A from *Pseudomonas aeruginosa* (EPA).^16,18−20^

*E. coli is* an opportunistic Gram-negative bacterium commonly found in the gut of humans and other warm-blooded animals. Commensal strains have a symbiotic relationship with the host while pathogenic strains can cause severe extra-intestinal diseases such as urinary tract infections, neonatal meningitis, and sepsis. Vaccines against extra-intestinal pathogenic *E. coli* (ExPEC) are needed to contrast the rising incidence of *E. coli* infections and the emergence of AMR strains.^21^ The high strain diversity of *E. coli* is shown by the ∼186 different O-types that have been identified from the detection of different O-PSs.^22−25^ The O-PS is a component of the lipopolysaccharide (LPS) that is present on the outer membrane of Gram-negative bacteria and is highly immunogenic. This characteristic has been successfully used for the development of bacterial vaccines.^16,20,26^

The bioconjugation of O-PSs to a carrier protein can lead to a highly heterogeneous glycoprotein. The biosynthesized polysaccharide is polydisperse, as it is constituted of a varying number of repeat units (RUs) formed of three to five monosaccharides. The polysaccharide length can vary considerably ranging up to tens of repeat units. The number of polysaccharide molecules conjugated to the carrier proteins also varies, depending on the number of possible conjugation sites. Consequently, the comprehensive analytical characterization of glycoconjugate bacterial vaccines, needed to corroborate their structural integrity, is not trivial and requires a multi-methods approach.^27^

Mass spectrometry (MS) is a powerful tool for the analysis of complex glycoproteins enabling the characterization of their amino acid sequence and glycosylation.^28^ Glycoproteins, i.e. proteins containing oligosaccharides, have been successfully characterized at different structural levels (i.e., released glycans, glycopeptides, and intact protein), whereas the MS analysis of bioconjugated proteins that contain O-PSs remains very challenging and is thus scarcely reported.^29−32^ Nevertheless, useful structural information has been obtained by MS from purified bacterial LPSs and O-PSs, often corroborating or complementing the information obtained by NMR spectroscopy.^33,34^ These molecules are difficult to ionize and often undergo fragmentation during the ionization process. Consequently, O-PSs have been rarely measured in their intact form. Instead, the analysis of their fragment ions has allowed the determination of the monosaccharide composition of the oligosaccharide repeat units.^35−37^ This approach can be used for the characterization of O-PSs of bioconjugated bacterial vaccines.

MALDI-in-source decay (ISD) is a fragmentation technique that can be applied to both intact proteins and polysaccharides.^38−40^ The combination of MALDI-ISD with ultrahigh-resolution FT-ICR MS enables the detailed analysis of both protein and carbohydrate fragment ions over an extended *m*/*z* range.^39,41,42^ Interestingly, MALDI-ISD of intact glycoproteins has only provided protein sequence information through the analysis of protein backbone fragment ions. To date, glycan information has only been obtained through the detection of glycosylated protein fragments (or their MS/MS analysis) and not from the direct detection of glycan fragments that were generated from in-source fragmentation of the glycan moieties from the proteins.^41,43^ MALDI analysis is fast and robust and does not require upfront separation techniques as often needed for ESI MS. MALDI is particularly suitable for high-throughput analyses and features short measurement times.

In this study, we propose a novel analytical strategy based on ultrahigh-resolution MALDI-ISD FT-ICR MS for the corroboration of both the amino acid sequence and the composition of the oligosaccharide repeat units of bioconjugated bacterial vaccines. Three different glycoconjugate vaccines, obtained from the bioconjugation of the O-PSs from *E. coli* serotypes O2, O6A, and O25B with EPA protein, were analyzed. ISD fragment ions from the protein backbone (i.e., c-, y-, and z-types) and the polysaccharide chains (i.e., B-, C-, Y-types) were selectively detected using 1,5-diaminonaphthalene and a 2,5-dihydroxybenzoic acid/2-hydroxy-5-methoxybenzoic acid mixture (super-DHB), respectively. The proposed analytical strategy shows potential for use in early and late development as well as quality control (QC), for example, for screening of glycoconjugate variants or for complementary analysis in a multi-methods approach.

## Experimental Section

### Samples

The three different glycoconjugate vaccines were obtained from the bioconjugation of the O-PSs from *E. coli* serotypes O2, O6A, and O25B with EPA protein, using recombinant *E. coli* production strains.^16,21^ The samples, EcoO2, EcoO6A, and EcoO25B, were provided by Janssen Vaccines AG (Branch of Cilag GmbH International), Bern, Switzerland. The bioproduction of the glycoconjugate vaccines was performed using Protein Glycan Coupling Technology (PGCT).^14^ Briefly, *E. coli* cells were bioengineered using plasmids for the expression of the carrier protein EPA, the O-antigen polysaccharides, and the oligosaccharyltransferase PgIB. In the cells, the synthesis of the polysaccharide repeat units (RUs) occurs at the cytoplasmic side on the inner membrane. They are built upon an undecaprenyl pyrophosphate lipid molecule carrier to form lipid-linked oligosaccharides (LLOs). The LLOs are then transferred to the periplasm side where they are recognized by PglB which transfers the polysaccharide chains onto acceptor sequons (D/E-X-N-X-S/T) on the carrier protein to produce the *N*-glycosylated glycoconjugate vaccine.

The glycoconjugates were separated from host-cell impurities by a series of conventional purification methods. All glycoconjugate vaccines were then subjected to a structural and compositional characterization at monosaccharide, intact protein, and glycopeptide levels.

### Buffer Exchange

The bioconjugates were provided in Bis-Tris/NaCl buffer. Prior to MALDI-ISD FT-ICR MS analysis of the carrier protein, samples were buffer-exchanged using reversed-phase solid-phase extraction (SPE). To this end, 2 μL of bioconjugate solution were added to 10 μL of water in a 500 μL Eppendorf tube. A C18-ZipTip (Merck Millipore) was washed three times with 15 μL of a water/ACN/formic acid solution (v/v, 50:49.95:0.05) and then conditioned three times with 15 μL of water. The diluted bioconjugate was allowed to bind to the SPE tip by pipetting 15 times up and down. Finally, the loaded SPE tip was flushed three times with 15 μL of water and the sample was eluted in 2 μL of water/ACN/formic acid (v/v, 50:49.95:0.05) directly onto a MALDI target plate.

### MALDI Sample Spotting

For glycan analysis, 1 or 2 μL of the glycoconjugate samples in their original buffer were spotted onto a ground-steel MALDI target plate with 1 μL of either a 10 mg/mL “super-DHB” (a 9:1 (w/w) mixture of 2,5-dihydroxybenzoic acid and 2-hydroxy-5-methoxybenzoic acid; purchased from Sigma-Aldrich, Germany) solution in 50:50 (v/v) ACN/water and NaOH 1 mM or a 100 mg/mL super-DHB solution in 50:50 (v/v) ACN/water. The spots were left to dry at room temperature. For the analysis of the protein backbone, the glycoconjugate samples were desalted by C18-ZipTip SPE as described above, by direct elution onto a polished-steel MALDI target plate and the addition of 1 μL of a saturated solution of 1,5-diaminonaphtalene (1,5-DAN) in water/ACN/formic acid (v/v, 50:49.95:0.05). The spots were left to dry at room temperature.

### MALDI-ISD FT-ICR Mass Spectrometry

MS measurements were performed on a 15 T solariX XR FT-ICR mass spectrometer (Bruker Daltonics, Bremen, Germany) equipped with a CombiSource and a ParaCell. The MS system was operated using ftmsControl software (Bruker Daltonics). MALDI measurements were performed using a Smartbeam-II laser system (Bruker Daltonics) at a frequency of 500 Hz and 200 laser shots per scan. Two acquisition methods, optimized for the detection of positive ions in the *m*/*z*-range 1000–7000 and negative ions in the *m*/*z*-range 300–5000, were used for the measurement of the protein ISD fragments with 1 M data points. Four acquisition methods were used for the analysis of the O-PS fragments. These methods were tuned for increased sensitivity in the *m*/*z*-ranges 3500–30000 (with 0.5 M data points), 300–8000 (with 1 M data points), and 1000–7000 (with 1 M data points) for MS measurements and 94–8000 (with 2 M data points) for MS/MS analysis. Collision-induced dissociation (CID) of carbohydrate fragment ions was performed on selected precursor ions. The collision energy was optimized for each precursor ion (40–80 V) for efficient fragmentation.

### Data Analysis

Theoretical fragment ions of EPA protein were generated using the online MS-product ProteinProspector tool (prospector.ucsf.edu/prospector/mshome.htm). Mass spectra were visually inspected using DataAnalysis 5.0 SR1 (Bruker Daltonics). Only fragment ions with a mass measurement error lower than 15 ppm were assigned. Negative fragment ions were assigned up to *m*/*z* 1500.

Theoretical fragment ions of the O-antigen oligosaccharide repeat units were generated in GlycoWorkbench 2.1 stable build 146 and matched with observed ions.^44,45^ The assignments were then corroborated by a visual inspection of the mass spectra.

## Results and Discussion

### N- and C-Terminal Sequencing of Bioconjugate *E. coli* Vaccines by MALDI-ISD FT-ICR MS

Carrier proteins are important components of glycoconjugate vaccines.^46^ Detoxified exotoxin A from *Pseudomonas aeruginosa* (EPA) has been used as a carrier protein in several immunization studies.^47−50,21^ In the bioconjugation process, EPA is recombinantly expressed and conjugated with O-PSs. This process can lead to proteoforms of EPA with different modifications of the amino acid sequence.^51^ Therefore, the structural integrity of the protein sequence must be verified to ensure the safety and quality of the bioconjugate product. Typically, protein sequence information can be obtained from the enzymatic digestion of glycoproteins and LC-MS/MS analysis of (glyco)peptides. Alternatively, proteins can be analyzed in their intact form using “top-down” MS-based strategies.^52^ One of these strategies involves the use of MALDI-ISD MS to generate protein fragment ions directly during the ionization process.^38,53^ Since MALDI primarily leads to singly charged ions, the comprehensive analysis of the generated ISD fragments requires a wide *m*/*z*-range of detection. Consequently, MALDI-ISD MS typically allows for the characterization of the terminal regions. Recently, we showed that ultrahigh-resolution MALDI-ISD FT-ICR MS, in both positive and negative ion modes, allows for the in-depth characterization of intact (glyco)proteins.^41,42,54,55^ Although MALDI is tolerant to the presence of a small amount of salt in the sample, desalting before MS analysis can significantly increase the quality of the ISD mass spectra of proteins.

**Figure 1:**
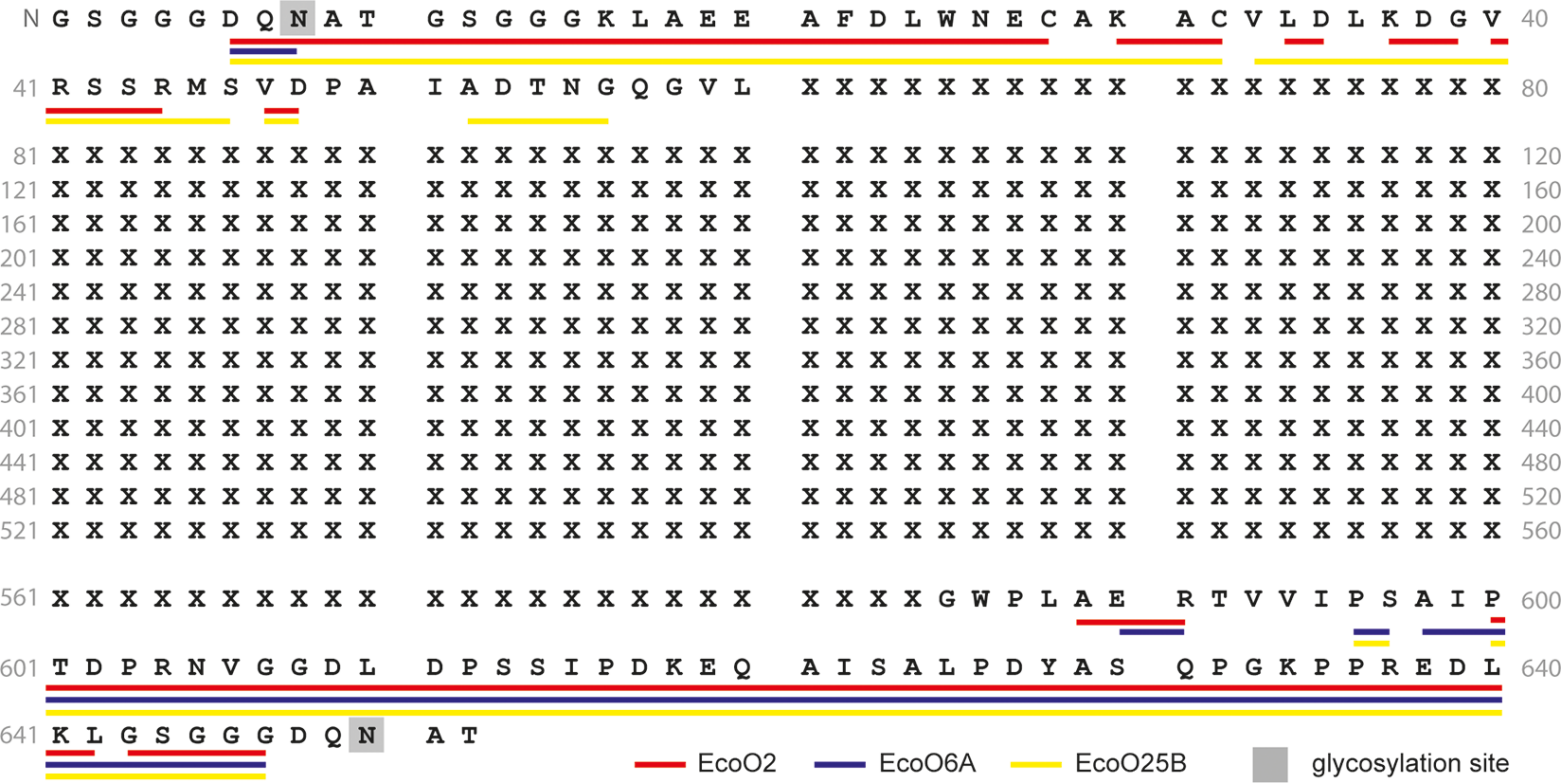
Sequence coverage of the carrier protein EPA obtained from the analysis of the glycoconjugates EcoO2, EcoO6A, and EcoO25B by MALDI-ISD FT-ICR MS (see also Figure S2 and Supporting Tables S1–S4). Only the sequence portions covered by the sequencing method are indicated.

In this study, we explored MALDI-ISD FT-ICR MS for the characterization of the glycoconjugates EcoO2, EcoO6A, and EcoO25B to confirm the N- and C-terminal portions of the EPA protein that was recombinantly expressed (Figures S1–S2). Typically, a long series of c′-, y-, and z′-type fragment ions were detected up to *m*/*z* 7000 and used for the determination of the sequence coverage which was 12%, 8%, and 14% for EcoO2, EcoO6A, and EcO25B, respectively (Figures 1 and S2 and Supporting Tables S2–S5). The presence of Bis-Tris and NaCl in the sample buffer affected the analysis, and desalting of the samples was therefore key for the sensitive detection of protonated or deprotonated protein fragment ions. Ultrahigh-resolution measurements allowed for a reliable identification of ISD fragment ions even in *m*/*z*-region below *m*/*z* 1000 which is typically dominated by MALDI matrix cluster ions. As previously shown for other proteins, analysis in negative ion mode improved the detection of small ISD fragment ions (Figure S3).^55^ Of note, for EcoO6A, the series of c′-type fragment ions was limited to c′6 and c′7 (Figures 1 and S4–S5). EPA contains four potential glycosylation sites, and only two of these (i.e., Asn8 and Asn650) are located close to the protein termini and are covered by our sequencing method. The absence of nonglycosylated *c*′-type fragment ions larger than *c*′7 observed for EcoO6A may be explained by the fact that Asn8 of this glycoconjugate vaccine is almost fully occupied, as is supported by glycopeptide analysis (data not shown). Glycosylated peptide fragment ions were not detected, possibly as a consequence of their high *m*/*z*-values and the low sensitivity at *m*/*z*-values higher than 7000. The first 5 amino acids from both protein termini were not directly characterized; however, the detection of the negative fragment ions c′6 (*m*/*z* 446.1641; sequence (N-terminus)-GSGGGD) and z′6 (*m*/*z* 588.2270; sequence GDQNAT-(C-terminus)) provides strong evidence of the integrity of the N- and C-terminal portions. A long series of z′-type ions were detected for all the glycoconjugates indicating a low glycosylation site occupancy at Asn650. These results, which were obtained from a fast analysis of the glycoconjugates and a straightforward interpretation of the mass spectra, provided useful information on the EPA protein N- and C-termini.

### Direct Analysis of the Oligosaccharide Repeat Units of *E. coli* Bioconjugate Vaccines by MALDI-ISD FT-ICR MS

The structural analysis of O-PSs conjugated to carrier proteins is pivotal for assessing the integrity of glycoconjugate bacterial vaccines, which is generally understood to be strongly related to their safety and efficacy. Bacterial O-PSs are usually constituted of large and polydisperse sequences of oligosaccharide subunits. Since MS analysis of intact O-PSs is challenging, fragment ions are commonly registered to study the monosaccharide composition of their repeat units.^35−37^ However, such a strategy is not commonly used for the analysis of glycoconjugate vaccines. Recently, we showed that ultrahigh-resolution MALDI FT-ICR MS allows for a detailed analysis of intact monodisperse synthetic polysaccharides and their ISD fragment ions which were produced using super-DHB as a MALDI matrix.^39,40^ The sensitive analysis of MALDI-ISD fragment ions of polysaccharides required the presence of cations (e.g., Na^+^). Therefore, in this study, the glycoconjugates EcoO2, EcoO6A, and EcoO25B were also analyzed by MALDI-ISD FT-ICR MS with super-DHB and without desalting of the samples which were in Bis-Tris/NaCl buffer. This allowed for the suppression of the ISD fragment ions generated from the protein moiety enhancing instead the signal of sodiated polysaccharide fragment ions.

**Figure 2:**
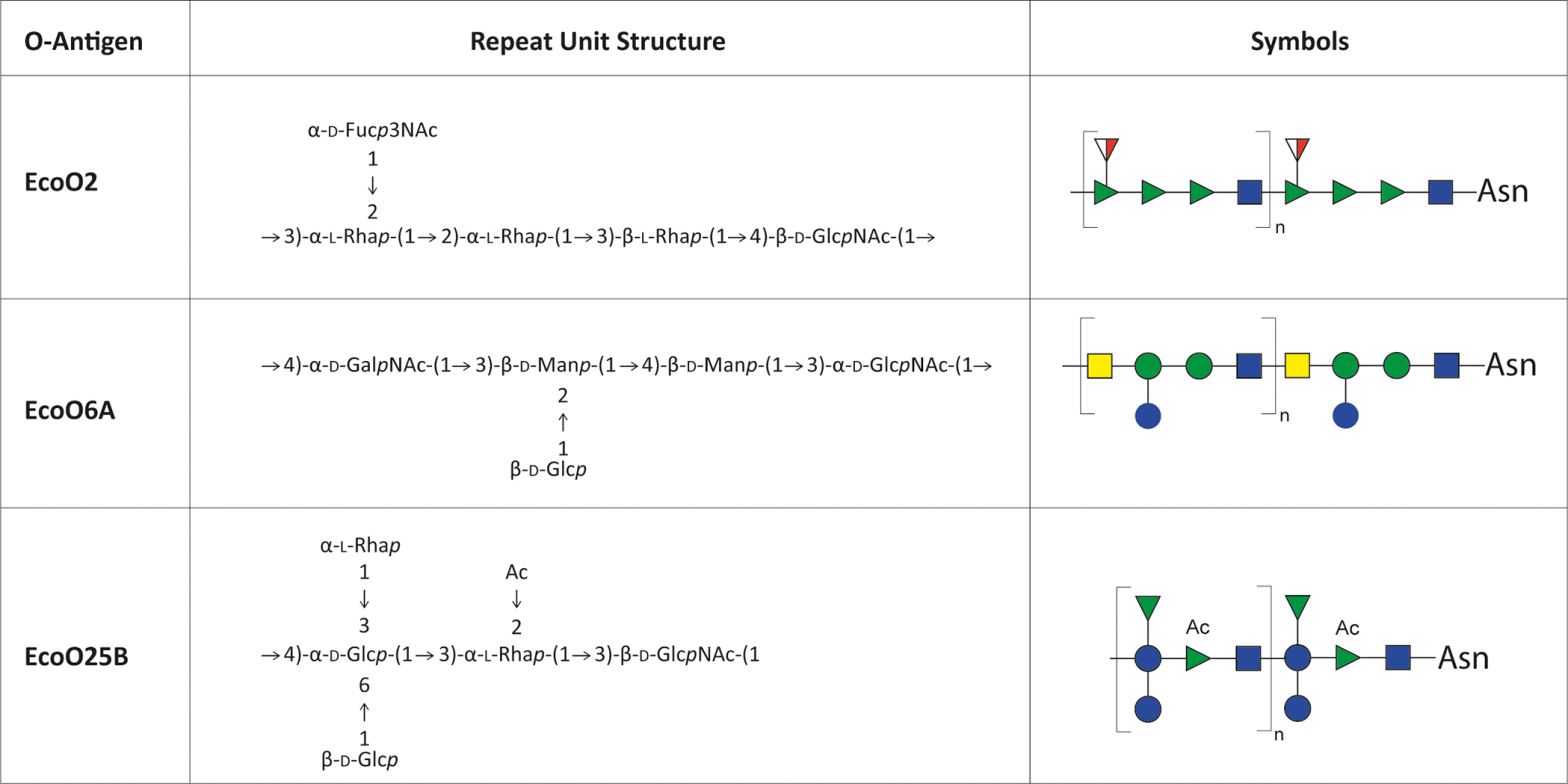
Structures and SNFC symbols of the O2, O6A, and O25B O-antigen oligosaccharide repeat units (RUs). These structures were previously reported.^56−59^d-Fuc3NAc is 3-acetamido-3-deoxy-d-fucose. Calculated mass of O2 RU (C_34_H_56_N_2_O_21_): 828.3376 Da. O6A RU (C_34_H_56_N_2_O_25_): 892.3172 Da. O25B RU (C_34_H_55_NO_24_): 861.3114 Da. The O-antigen polysaccharides are directly linked to Asn residues on the carrier protein. SNFC symbols: rhamnose, green triangle; *N*-acetylglucosamine, blue square; *N*-acetylfucosamine, white/red triangle; *N*-acetylmannosamine, yellow square; glucose, blue circle; mannose, green circle; acetylation, Ac.

The structure of the oligosaccharide repeat units of *E. coli* serotypes O2, O6A, and O25B have been previously elucidated and reported (Figure 2).^56−59^

**Figure 3:**
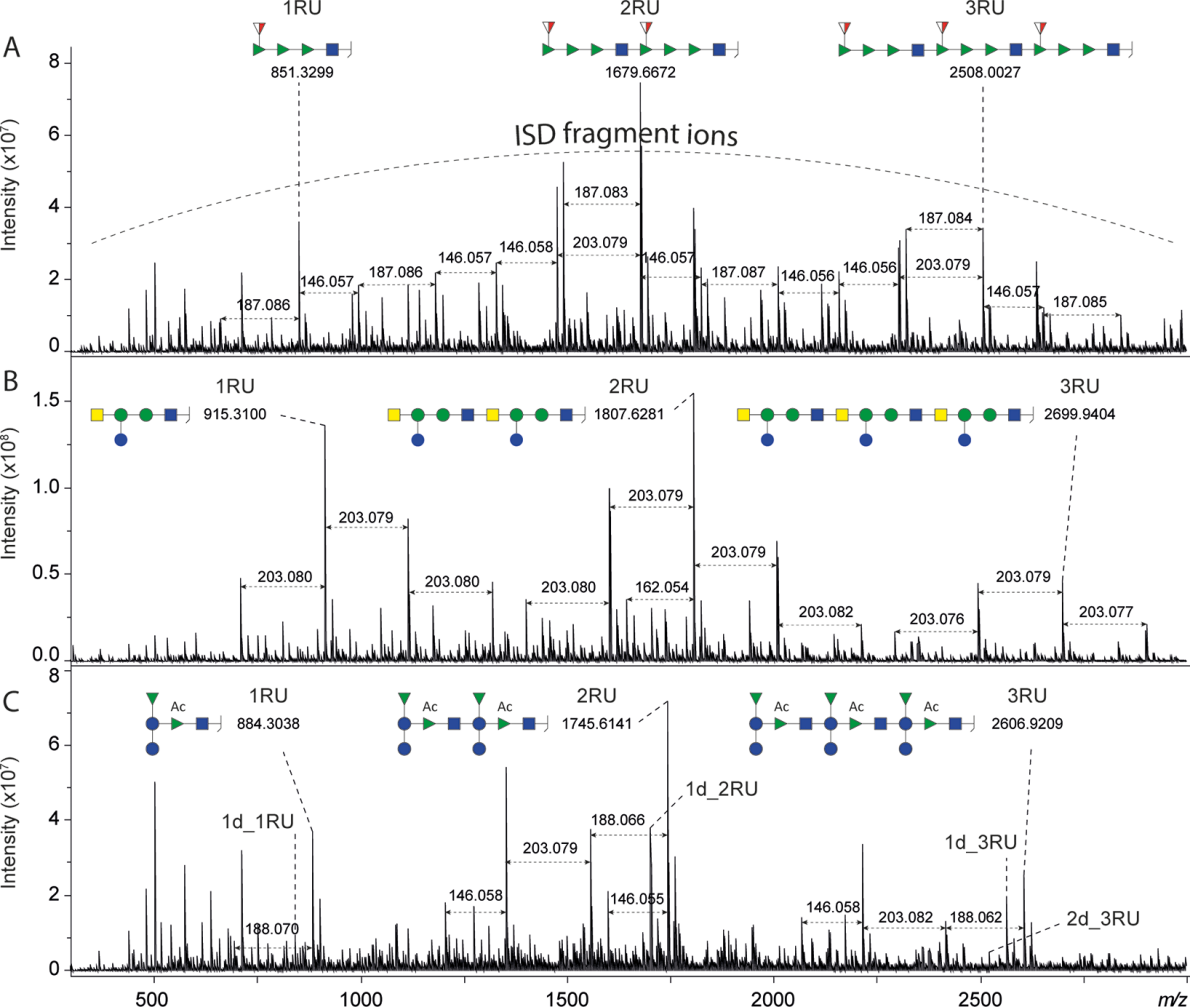
Enlargements of MALDI-ISD FT-ICR mass spectra of the glycoconjugates EcoO2 (A), EcoO6A (B), and EcoO25B (C) in the *m*/*z*-range 300–3000. Sodiated B ions corresponding to one, two, and three repeat units (1RU, 2RU, and 3RU) were detected and annotated. Not annotated peaks were generated from the cleavage of different glycosidic bonds as exemplified in Figure 4 and Figures S9–S10 for the *m*/*z*-range between 1RU and 2RU. Compositional differences between these fragment ions are exemplified by the reported *m*/*z* differences. The theoretical monosaccharide residual masses are the following: hexose = 162.0528 Da; deoxyhexose = 146.0579 Da; *N*-acetylhexosamine = 203.0794 Da; *N*-acetyldeoxyhexosamine = 187.0845 Da; acetylated deoxyhexose = 188.0685 Da. The calculated *m*/*z*-values of sodiated B-ions corresponding to one, two and three RUs are reported in Table S5.

**Figure 4:**
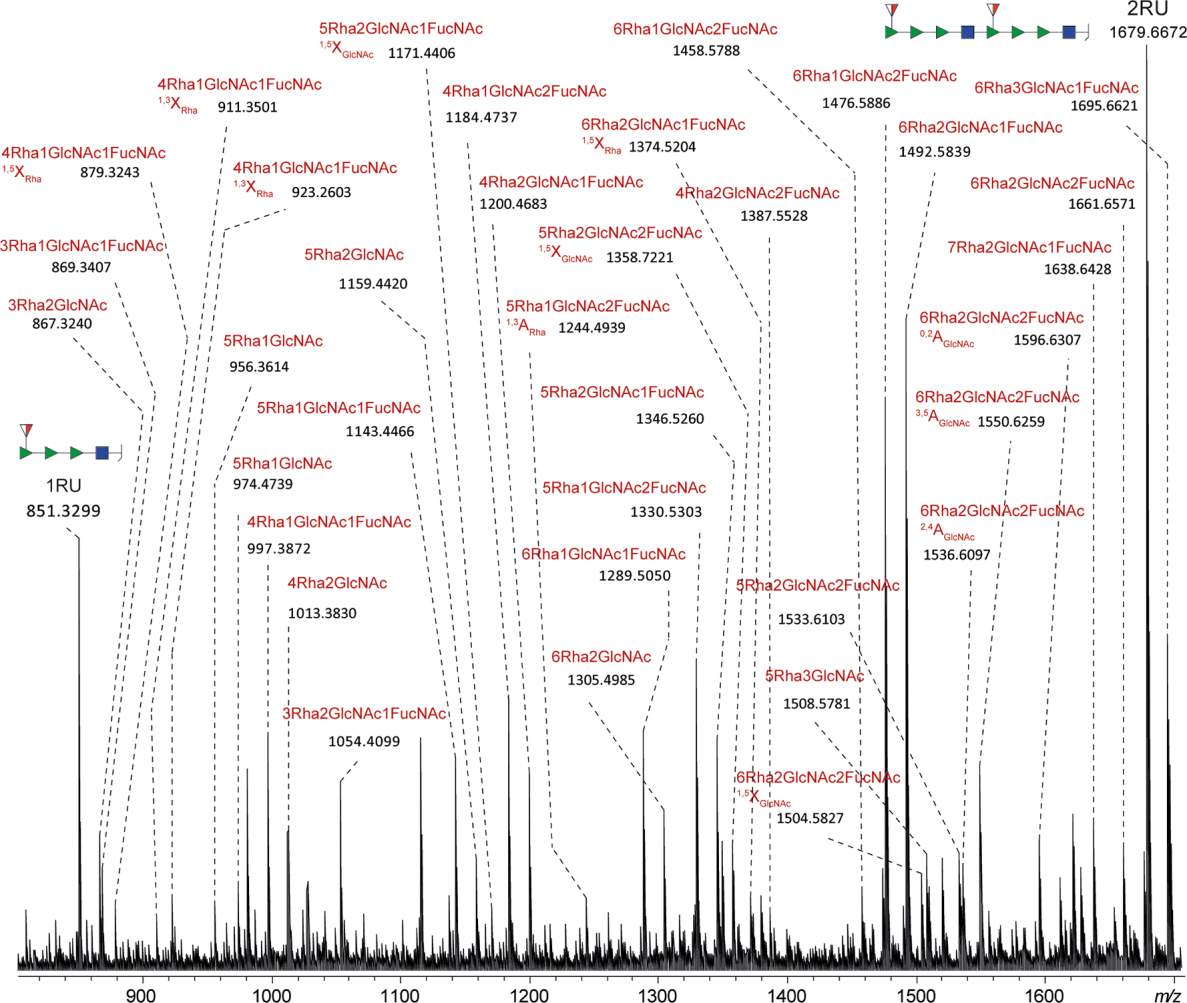
Enlargement of the MALDI-ISD FT-ICR mass spectrum of the glycoconjugate EcoO2 (see Figure 3) in the *m*/*z* range between sodiated B-ions corresponding to 1RU and 2RU. The cleavage of one or more glycosidic bonds led to the formation of the fragment ions highlighted in red. Different isomeric fragment ions may exist for each assigned peak.

Thus, this study aimed to assess the applicability of MALDI-ISD FT-ICR MS for a straightforward corroboration of the O-antigen compositions and structures directly from the analysis of the bioconjugate vaccines. The MALDI-ISD FT-ICR mass spectra, in the *m*/*z*-range 300–3000, of the three glycoconjugates are reported in Figure 3. The fragmentation process led to the cleavage of the glycosidic bonds (from any of the different polysaccharide chains attached to the EPA protein) and the formation of sodiated B and C fragment ions with B ions being more intense (Figure S6). B ions covering a single repeat unit (named 1RU) were detected at *m*/*z* 851.3299, 915.3100, and 884.3038, for EcoO2, EcoO6A, and EcoO25B, respectively. B ions corresponding to two (2RU) and three repeat units (3RU) were also observed in the same mass spectrum while larger B ions corresponding to up to 11 repeat units could be detected using different acquisition methods and depending on the analyzed glycoconjugate (Figures S7–S8). Of note, the number of repeat units on a polysaccharide chain could be higher than what was detected by MALDI-ISD FT-ICR MS as larger fragment ions may suffer from a lower ionization efficiency, and measurements at high *m*/*z*-values are notoriously less sensitive. In addition to the fragment ions corresponding to complete repeat units, more fragment ions were detected as a consequence of the cleavage of one or more different glycosidic bonds (Figures 3–4 and S9–S10). The mass spectra were further complicated by a high chemical background derived from the analysis of not desalted samples. The evaluation of the mass defect (i.e., the difference between exact and nominal mass) allowed for the reliable identification and exclusion of these background peaks.

For EcoO25B, a certain degree of deacetylation of the repeat units was expected as the glycopeptide analysis (data not shown) revealed an average number of acetylation per repeat unit of about 0.5. The presence of acetylation variants introduced a higher degree of complexity in the mass spectra (Figure S10). The ratios between deacetylated (d_RU) and acetylated RU were determined for fragment ions corresponding to 1RU, 2RU, and 3RU (Figure 3C): 1d_1RU/1RU was 0.23; 1d_2RU/2RU was 0.53; 2d_2RU/2RU was 0.08; 1d_3RU/3RU was 0.73; 2d_3RU/3RU was 0.20. This corresponds to an average number of acetylation per repeat unit of about 0.8. The degree of deacetylation was higher for fragment ions corresponding to a higher number of RUs (Figure S7C).

Of note, the analysis of the polysaccharide moieties by MALDI-ISD FT-ICR MS, solely, may not be sufficient to exclude the presence of unexpected low-abundance polysaccharide variants; therefore, the use of other analytical strategies is recommended for a multi-level characterization of glycoconjugate bacterial vaccines.

**Figure 5:**
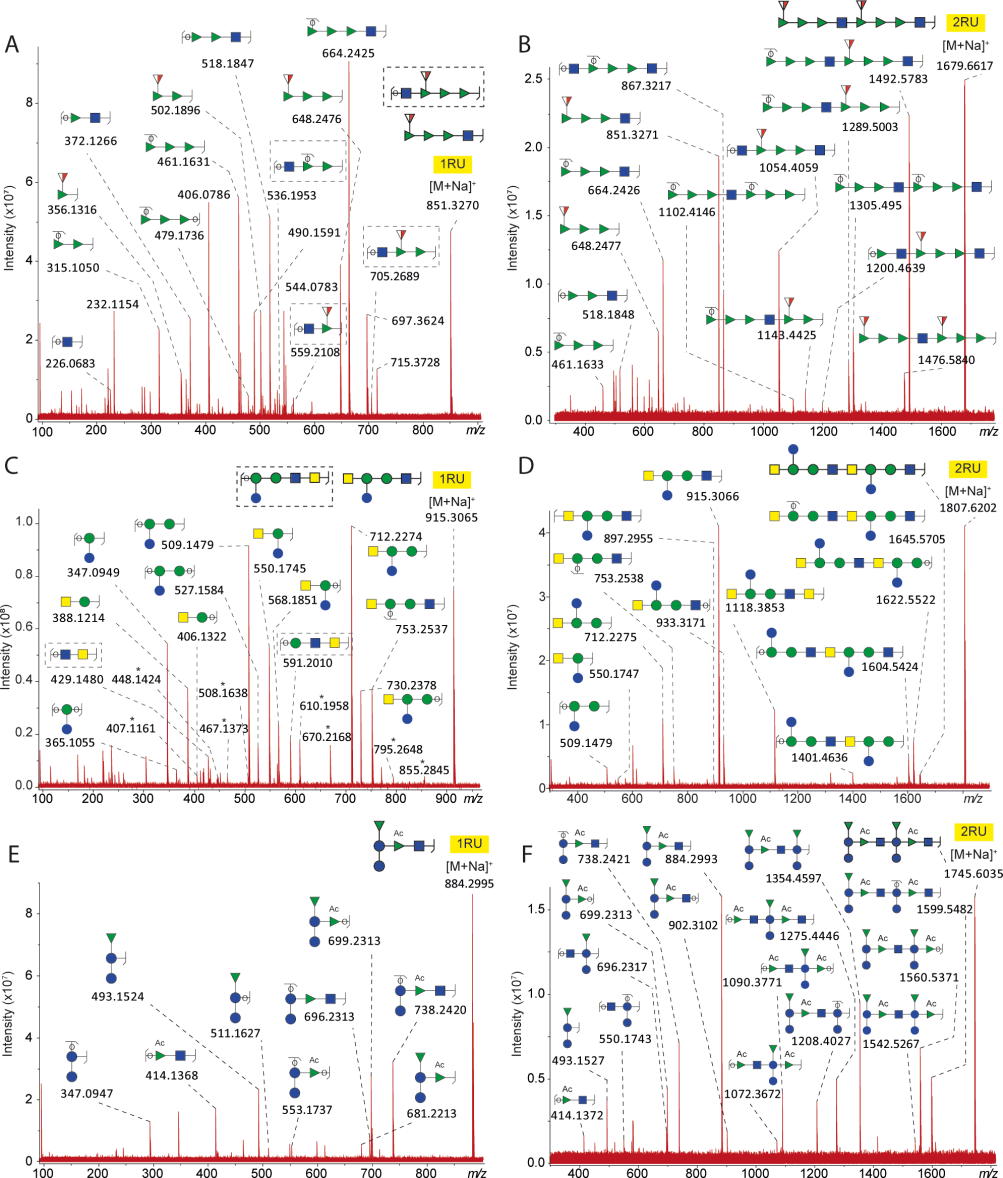
MALDI-ISD CID FT-ICR tandem mass spectra of the sodiated B fragment ion corresponding to 1RU and 2RU of the glycoconjugates EcoO2 (A and B), EcoO6A (C and D), and EcoO25B (E and F). For many species, several isomeric structures are possible of which only a single one is displayed. *Cross-ring fragment ions.

B ions corresponding to 1RU and 2RU were further characterized by CID MS/MS for corroboration of the repeat unit composition. The results of these analyses are reported in Figure 5. CID of the precursor ions may lead to isomeric fragment ions which cannot be distinguished in the mass spectra. For example, the fragment at *m*/*z* 1492.5783, detected in the CID mass spectrum of 2RU of EcoO2 (Figure 5B), was generated from the loss of one Fuc3NAc residue. Although this loss can occur at two positions, the figure was simplified by reporting only one structure. CID analysis also revealed the presence of isobaric ISD fragment ions generated from a double fragmentation of the polysaccharide chain. For example, two isomeric structures were assigned to the fragment ion detected at *m*/*z* 851.3270 in the CID mass spectrum of 1RU of EcoO2 (Figure 5A). One corresponded to the B ion of a single oligosaccharide repeat unit while the other was identified as a fragment ion generated from Y and B cleavages. Furthermore, the CID analysis of the ISD fragments generated from EcoO25B showed a diagnostic fragment ion at *m*/*z* 414.1368 confirming the position of the acetylated rhamnose (Figure 5B). Cross-ring fragment ions were solely detected in the mass spectrum generated from CID of 1RU of EcoO6A.

## Conclusion

Glycoconjugate bacterial vaccines, obtained from the bioconjugation of O-PSs and a carrier protein, commonly have a high structural heterogeneity that makes their analytical characterization challenging. In this study, for the first time, we applied ultrahigh-resolution MALDI-ISD FT-ICR MS for the characterization of three glycoconjugate bacterial vaccines constituted of *E. coli* O2, O6A, and O25B O-PSs and EPA as a carrier protein with the aim of a fast corroboration of specific structural features. Both the terminal amino acid sequence and the monosaccharide composition of the oligosaccharide repeat units were determined in a straightforward manner using the same MS-based method and only changing the sodium content in the samples and using either 1,5-DAN or super-DHB as a MALDI matrix. Of note, the generation of glycan MALDI-ISD fragment ions directly from intact glycoproteins was remarkable, as this analysis previously has typically provided protein and glycan information through the generation of protein backbone fragment ions (either glycosylated or not), solely.

CID MS/MS analysis of ISD fragment ions corresponding to one or two repeat units was used to confirm the oligosaccharide repeat structures. The use of MALDI allowed for fast analysis of the glycoconjugate vaccines without the need for separation instrumentation (e.g., liquid chromatography systems).

We envision the use of our new analytical strategy in a multi-methods approach for the assessment of the structural integrity of recombinant glycoconjugate bacterial vaccines. The fast determination of key structural features of carrier proteins and O-PSs by ultrahigh-resolution MALDI-ISD FT-ICR MS may find applications in the manufacturing process of recombinant glycoconjugate bacterial vaccines, from development phases (e.g., screening of glycoconjugate variants) to quality control (QC). Further investigations are required to evaluate the adaptation of this technology to other MALDI MS platforms that are more accessible to the biopharma industry for the analysis of recombinant glycoconjugate bacterial vaccines.

## Supporting Information Available

Additional details of MALDI-ISD FT-ICR mass spectra of the analyzed glycoconjugates; Schematic representation of the standardized nomenclature of fragment ions of carbohydrates. (PDF)
Tables with theoretical and observed ISD fragment ions. (XLSX)
