# Supplementary material for: Glycan and Protein Analysis of Glycoengineered Bacterial E. coli Vaccines by MALDI-in-Source Decay FT-ICR Mass Spectrometry
Source: Anal Chem. 2022 Mar 16;94(12):4979–87. doi: 10.1021/acs.analchem.1c04690 (PMC8969423; doi:10.1021/acs.analchem.1c04690)
Supplement: Supplementary file 1 — ac1c04690_si_001.pdf [file ac1c04690_si_001.pdf]

Supporting Information

Glycan and protein analysis of glycoengineered bacterial *E. coli* vaccines by MALDI-in-source decay FT-ICR mass spectrometry

Simone Nicolardi[*a], Renzo Danuser[b], Viktoria Dotz[#c], Elena Domínguez-Vega[a], Ali Al Kaabi[b], Michel Beurret[c], Chakkumkal Anish[c], and Manfred Wuhrer[a]

[a] Center for Proteomics and Metabolomics, Leiden University Medical Center, Albinusdreef 2, 2333 ZA Leiden, The Netherlands

[b] Janssen Vaccines AG (Branch of Cilag GmbH International), Rehhagstrasse 79, CH-3018 Bern, Switzerland

[c] Bacterial Vaccine Discovery & Early Development, Janssen Vaccines and Prevention B.V., Archimedesweg 4-6, 2333 CN Leiden, The Netherlands

*Corresponding author: Simone Nicolardi

Center for Proteomics and Metabolomics, Leiden University Medical Center, Albinusdreef 2, 2333 ZA Leiden, The Netherlands. E-mail: s.nicolardi@lumc.nl

[#]Current address: BioTherapeutics Analytical Development, Janssen Biologics B.V., Einsteinweg 101, 2333 CB Leiden, The Netherlands

Table of Content

Name	Description	Page #
Figure S1	MALDI-ISD FT-ICR mass spectra of glycoconjugate EcoO25B acquired in positive and negative ion modes	S3
Figure S2	Enlargements of the MALDI-ISD FT-ICR mass spectrum of glycoconjugate EcoO25B depicted in Figure S1A	S4
Figure S3	Examples of fragment ions detected in positive and negative ion modes	S5
Figure S4	Enlargements of negative mode MALDI-ISD FT-ICR mass spectra of the glycoconjugates EcoO2, EcoO6A, and EcoO25B	S7
Figure S5	Enlargements of negative mode MALDI-ISD FT-ICR mass spectra of the glycoconjugates EcoO2, EcoO6A, and EcoO25B	S7
Figure S6	Schematic representation of the standardized nomenclature of fragment ions of carbohydrates	S8
Figure S7	Enlargement of the MALDI-ISD FT-ICR mass spectra of the glycoconjugates EcoO2, EcoO6A, and EcoO25B in the *m/z*-range 3000-8000	S9
Figure S8	Enlargement of the MALDI-ISD FT-ICR mass spectrum of the glycoconjugate EcoO6A in the *m/z*-range 4000-12000	S10
Figure S9	Enlargement of the MALDI-ISD FT-ICR mass spectrum of the glycoconjugate EcoO6A in the *m/z*-range between 1RU and 2RU	S11
Figure S10	Enlargement of the MALDI-ISD FT-ICR mass spectrum of the glycoconjugate EcoO25B in the *m/z*-range between 1RU and 2RU	S12

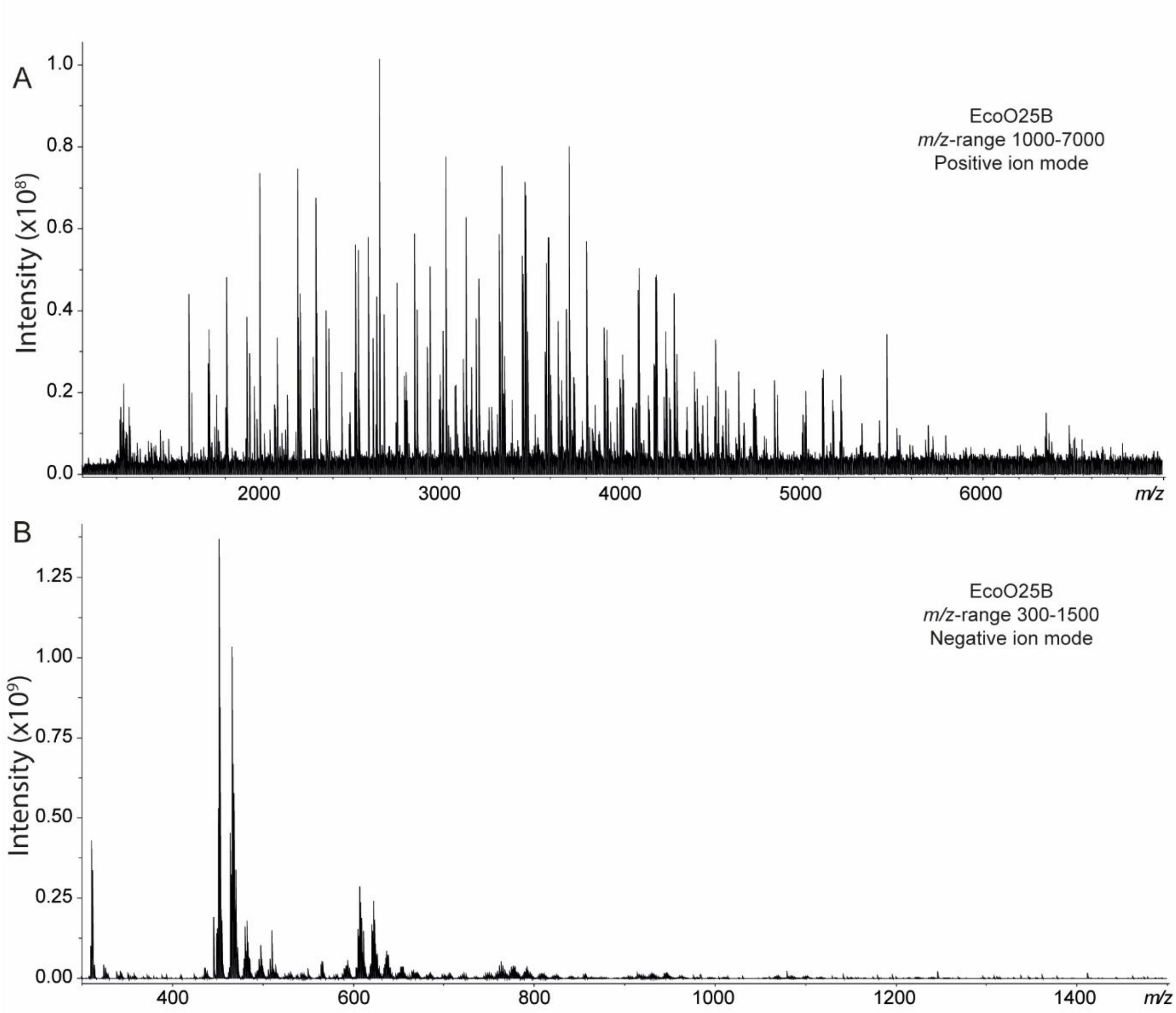

Figure S1. Examples of MALDI-ISD FT-ICR mass spectra acquired in (A) positive and (B) negative ion modes after desalting of the glycoconjugate EcoO25B. Such spectra were visually investigated and the signals matching the theoretical *m/z*-values of ISD fragment ions of EPA protein were assigned (see Figure S2). The lists of the assigned fragment ions are reported in Table S4. Enlargements of the spectrum depicted in A are reported in Figures S2. The spectrum depicted in B is characterized by the presence of intense MALDI ion clusters that are typically detected below *m/z* 1000. Ultrahigh-resolution measurements allowed for confident assignment in this region.

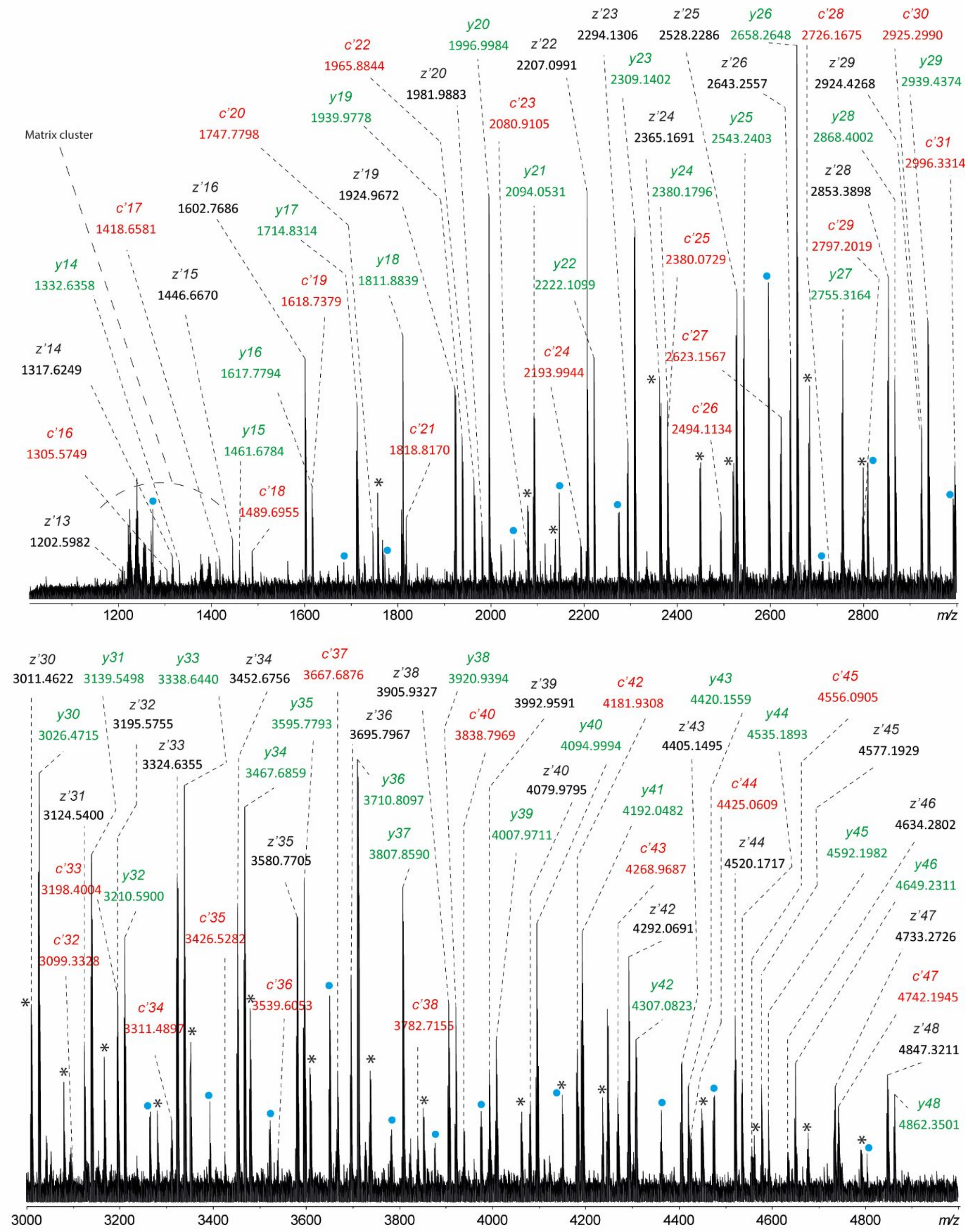

Figure S2. Enlargements of the MALDI-ISD FT-ICR mass spectrum of glycoconjugate EcoO25B depicted in Figure S1A. Detected *c’*-, *y*- and *z’*-type fragment ions were compared with theoretical values and assigned within a mass measurement error of 15 ppm. The assigned fragments are reported in Table S4. * indicates z’+1,5-DAN ions. Blue circle indicates *w*-type ions.

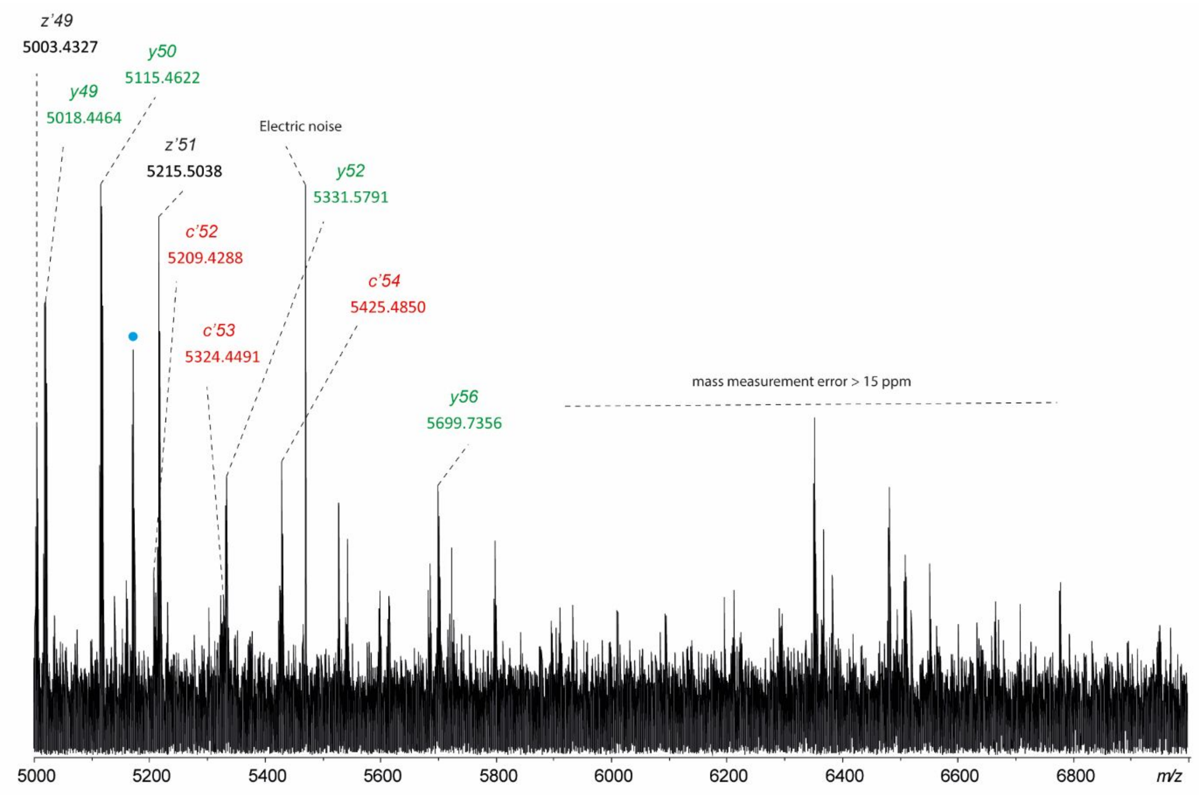

Figure S2. Continued

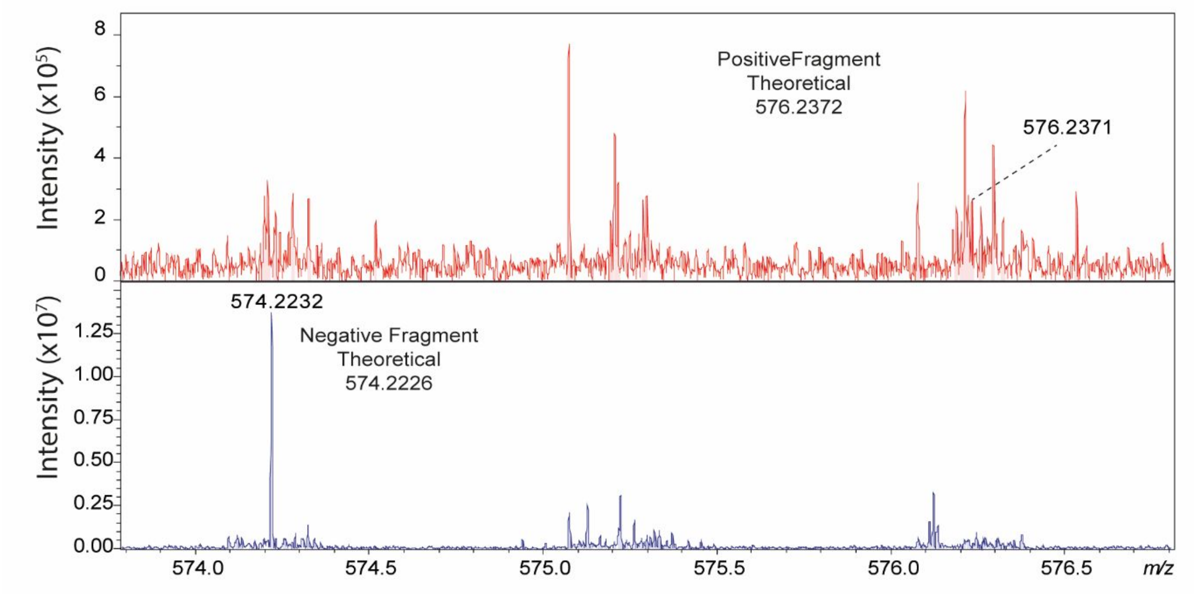

Figure S3. Examples of fragment ions detected in positive and negative ion modes. As previously shown (Nicolardi *et al.*, *Anal. Chem.* 2020, 92, 18, 12429–12436), negative mode MALDI-ISD FT-ICR MS can provide a higher signal intensity of fragment ions in the *m/z*-region of the MALDI matrix, i.e. typically below *m/z* 1000.

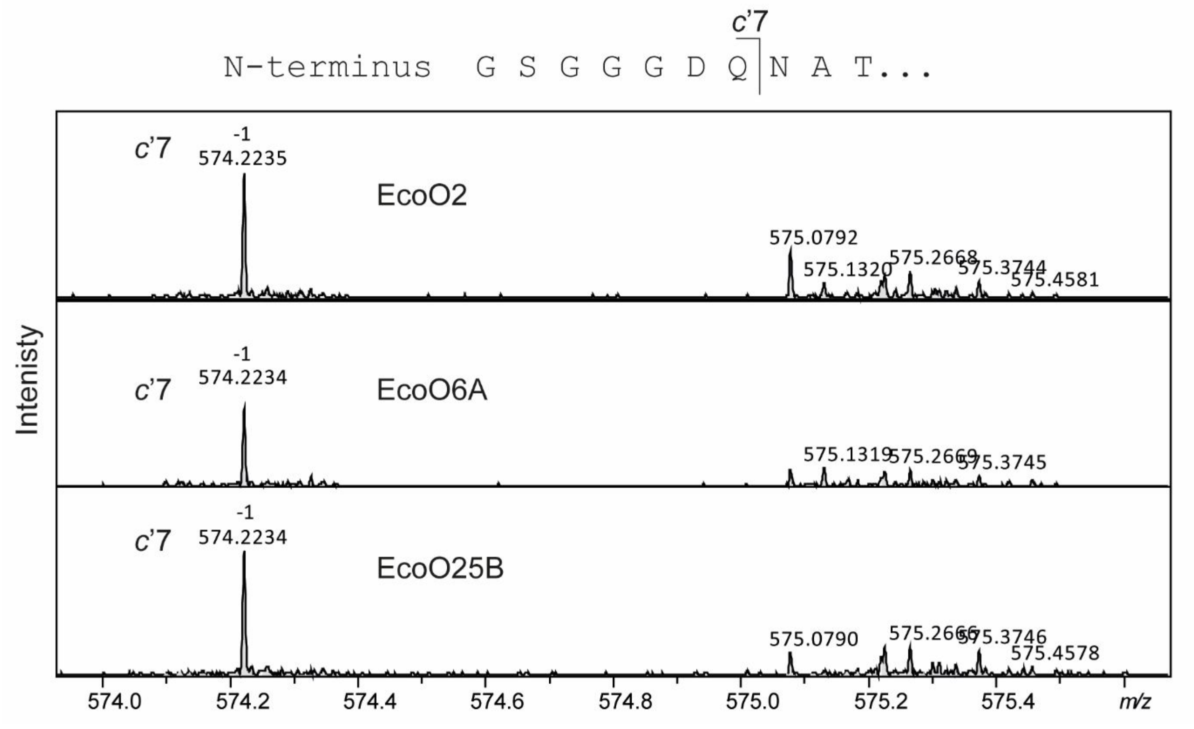

Figure S4. Enlargements of negative mode MALDI-ISD FT-ICR mass spectra of the glycoconjugates EcoO2, EcoO6A, and EcoO25B. The c'7 fragment ion, generated from the cleavage of the peptide bond between Gln7 and the potentially glycosylated Asn8, was detected in all mass spectra.

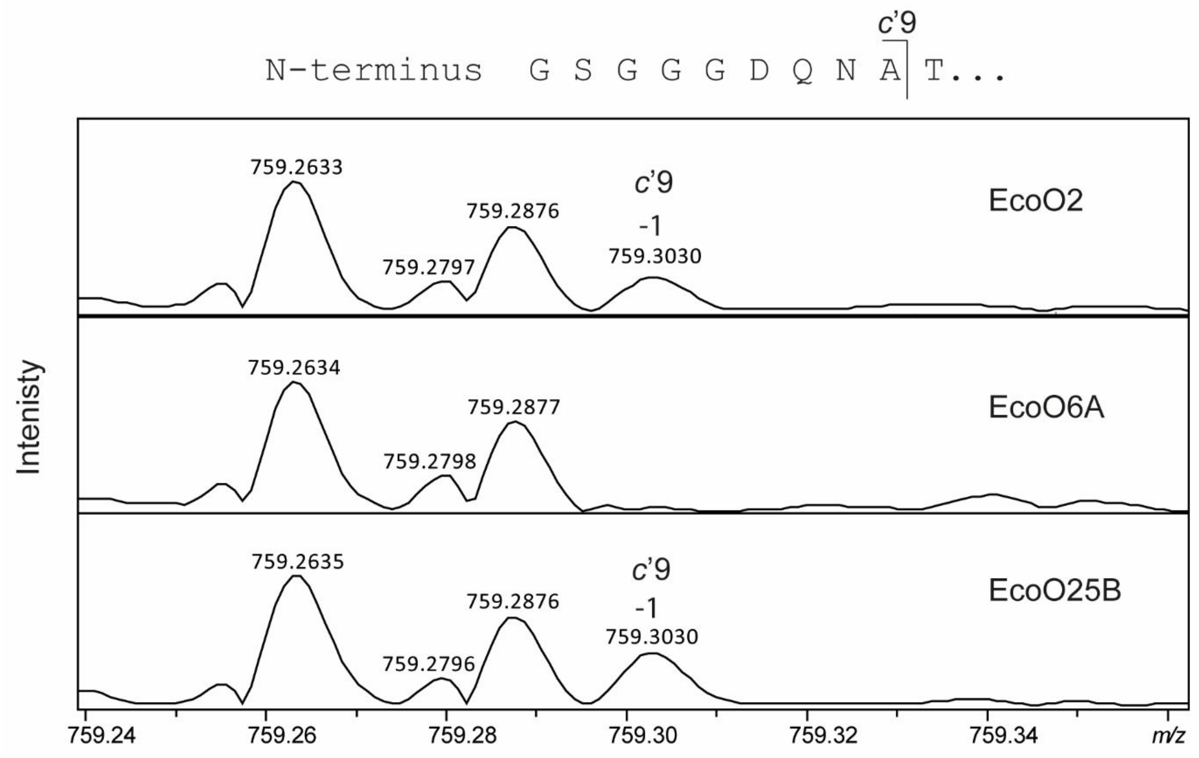

Figure S5. Enlargements of negative mode MALDI-ISD FT-ICR mass spectra of the glycoconjugates EcoO2, EcoO6A, and EcoO25B. The c'9 fragment ion included the potentially glycosylated Asn8. This fragment ion, as well as larger *c*'-type ions, was not detected in the mass spectrum of EcoO6A indicating a high glycosylation site occupancy at Asn8.

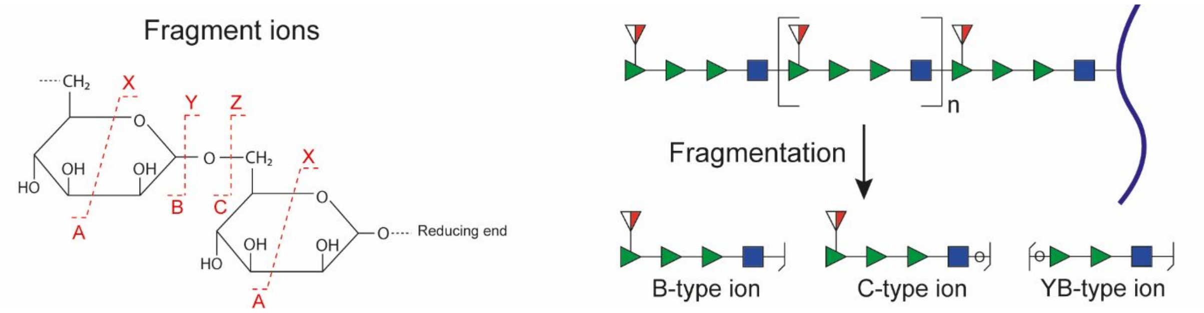

Figure S6. Schematic representation of the nomenclature of fragment ions of carbohydrates according to Domon and Costello (Domon, B.; Costello, C. E., Glycoconj. J. 1988, 5 (4), 397-409).

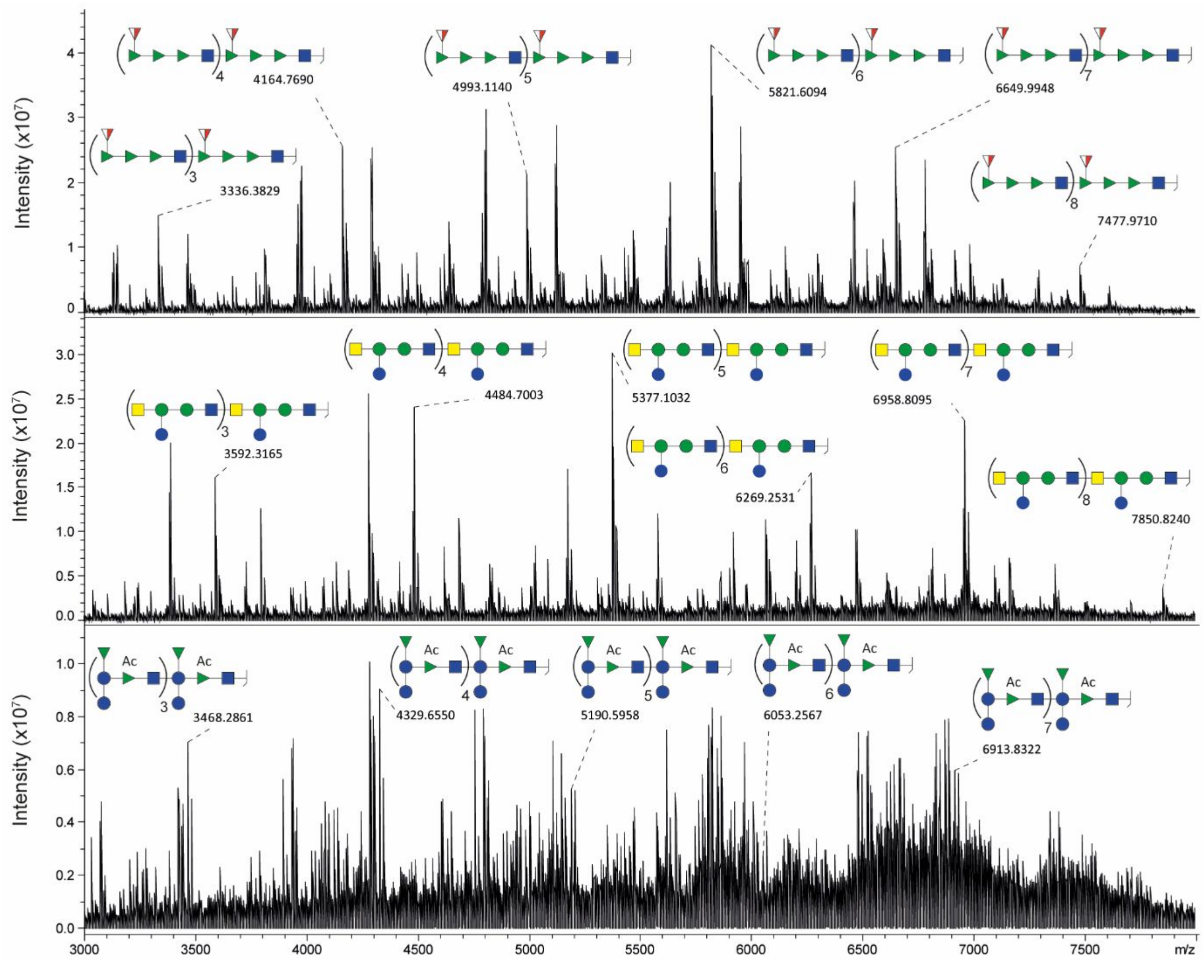

Figure S7. Enlargement of the MALDI-ISD FT-ICR mass spectra of the glycoconjugates EcoO2, EcoO6A, and EcoO25B in the *m/z*-range 3000-8000. Sodiated B ions corresponding to up to 9 repeating units were detected in this *m/z*-range. The mass spectrum of EcoO25B showed a higher complexity due to the presence of fragment ions generated from the partial loss of acetylation and C-type fragmentation. Not annotated peaks are fragment ions generated from the cleavage of one or more different glycosidic bonds (See Figures 4 and S9-S10).

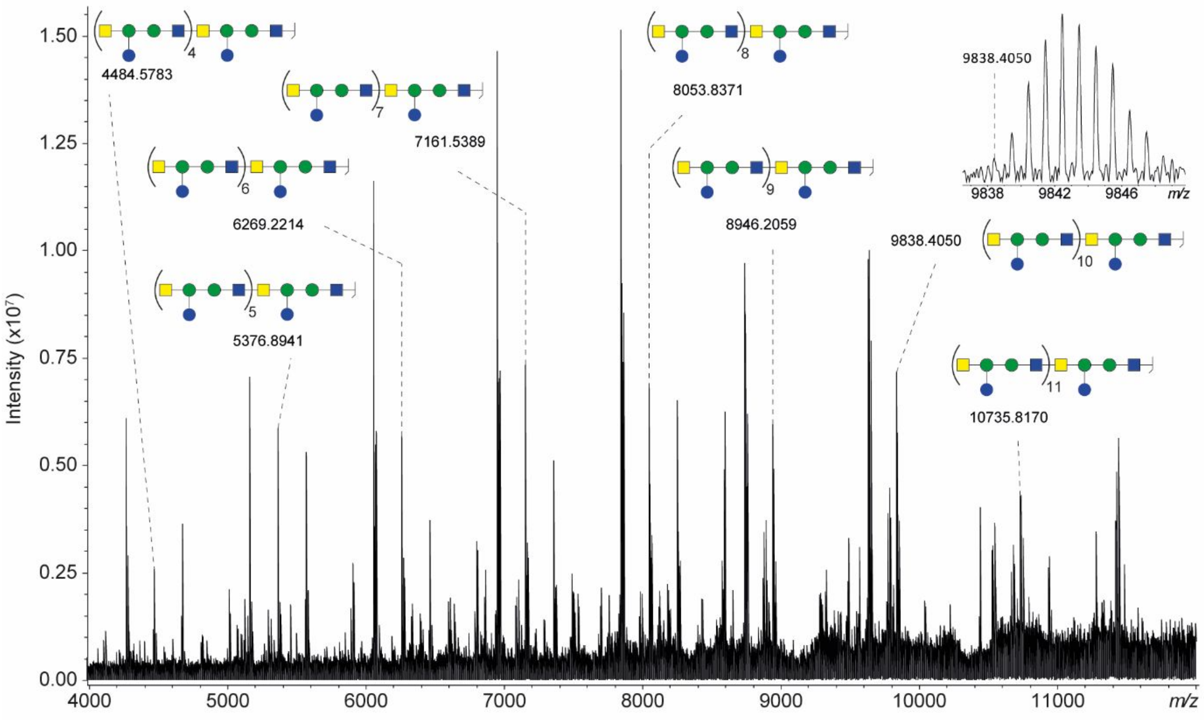

Figure S8. Enlargement of the MALDI-ISD FT-ICR mass spectrum of the glycoconjugate EcoO6A in the *m/z*-range 4000-12000. Sodiated B ions corresponding to up to 11 repeating units were detected in this *m/z*-range. Not annotated peaks are B (and C) ions generated from the cleavage of one or more different glycosidic bonds (see Figures 3 and S9-S10).

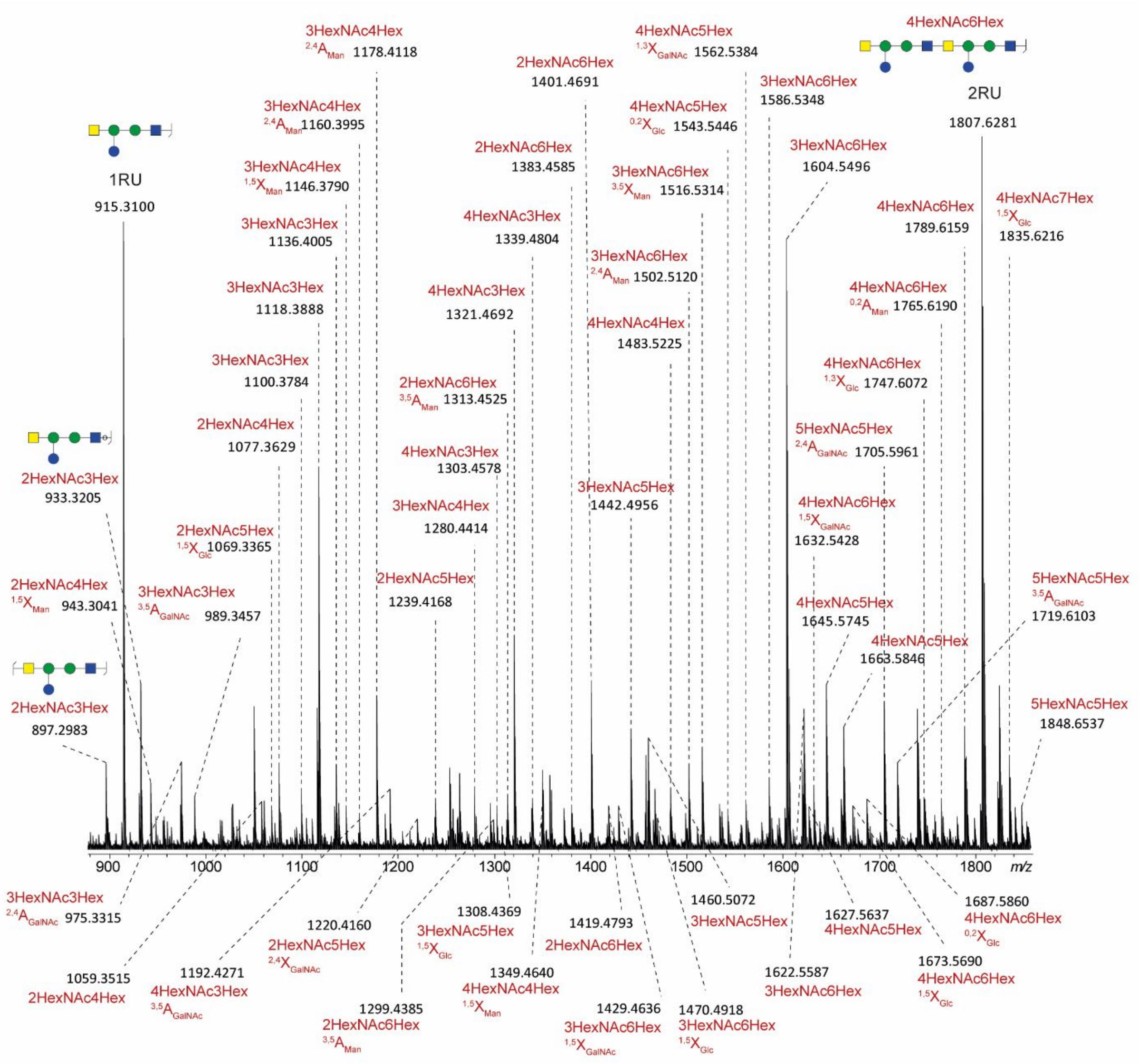

Figure S9. Enlargement of the MALDI-ISD FT-ICR mass spectrum of the glycoconjugate EcoO6A (see **Figure 3**) in the *m/z*-range between sodiated B-ions corresponding to 1RU and 2RU. The cleavage of one or more glycosidic bonds led to the formation of the fragment ions highlighted in red. Different isomeric fragment ions may exist for each assigned peak.

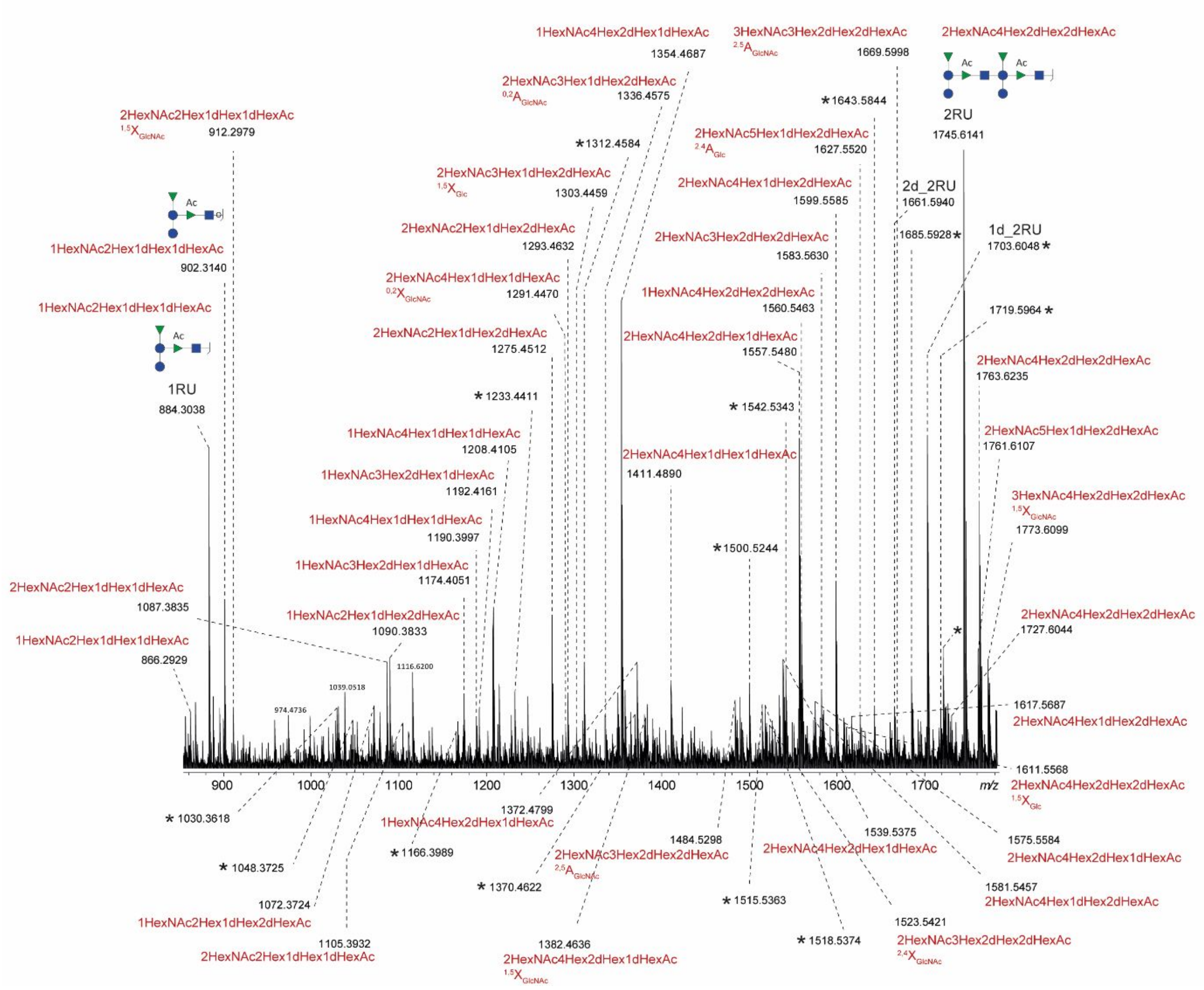

Figure S10. Enlargement of the MALDI-ISD FT-ICR mass spectrum of the glycoconjugate EcoO25B (see **Figure 3**) in the *m/z*-range between sodiated B-ions corresponding to 1RU and 2RU. The cleavage of one or more glycosidic bonds led to the formation of the fragment ions highlighted in red. Different isomeric fragment ions may exist for each assigned peak. * indicates fragment ions that were generated from repeat unit variants lacking one (or more) acetyl groups.
